# The effect of BCG revaccination on all-cause mortality beyond infancy: 30-year follow-up of a population-based, double-blind, randomised placebo-controlled trial in Malawi

**DOI:** 10.1016/S1473-3099(20)30994-4

**Published:** 2021-11

**Authors:** Judith R Glynn, Albert Dube, Katherine Fielding, Amelia C Crampin, Karonga Prevention Trial Group, Chifundo Kanjala, Paul E M Fine

**Affiliations:** a Faculty of Epidemiology and Population Health, London School of Hygiene & Tropical Medicine, London, UK; b Malawi Epidemiology and Intervention Research Unit, Chilumba and Lilongwe, Malawi

## Abstract

**Background:**

Trials of BCG vaccination to prevent or reduce severity of COVID-19 are taking place in adults, some of whom have been previously vaccinated, but evidence of the beneficial, non-specific effects of BCG come largely from data on mortality in infants and young children, and from in-vitro and animal studies, after a first BCG vaccination. We assess all-cause mortality following a large BCG revaccination trial in Malawi.

**Methods:**

The Karonga Prevention trial was a population-based, double-blind, randomised controlled in Karonga District, northern Malawi, that enrolled participants between January, 1986, and November, 1989. The trial compared BCG (Glaxo-strain) revaccination versus placebo to prevent tuberculosis and leprosy. 46 889 individuals aged 3 months to 75 years were randomly assigned to receive BCG revaccination (n=23 528) or placebo (n=23 361). Here we report mortality since vaccination as recorded during active follow-up in northern areas of the district in 1991–94, and in a demographic surveillance follow-up in the southern area in 2002–18. 7389 individuals who received BCG (n=3746) or placebo (n=3643) lived in the northern follow-up areas, and 5616 individuals who received BCG (n=2798) or placebo (n=2818) lived in the southern area. Year of death or leaving the area were recorded for those not found. We used survival analysis to estimate all-cause mortality.

**Findings:**

Follow-up information was available for 3709 (99·0%) BCG recipients and 3612 (99·1%) placebo recipients in the northern areas, and 2449 (87·5%) BCG recipients and 2413 (85·6%) placebo recipients in the southern area. There was no difference in mortality between the BCG and placebo groups in either area, overall or by age group or sex. In the northern area, there were 129 deaths per 19 694 person-years at risk in the BCG group (6·6 deaths per 1000 person-years at risk [95% CI 5·5–7·8]) versus 133 deaths per 19 111 person-years at risk in the placebo group (7·0 deaths per 1000 person-years at risk [95% CI 5·9–8·2]; HR 0·94 [95% CI 0·74–1·20]; p=0·62). In the southern area, there were 241 deaths per 38 399 person-years at risk in the BCG group (6·3 deaths per 1000 person-years at risk [95% CI 5·5–7·1]) versus 230 deaths per 38 676 person-years at risk in the placebo group (5·9 deaths per 1000 person-years at risk [95% CI 5·2–6·8]; HR 1·06 [95% CI 0·88–1·27]; p=0·54).

**Interpretation:**

We found little evidence of any beneficial effect of BCG revaccination on all-cause mortality. The high proportion of deaths attributable to non-infectious causes beyond infancy, and the long time interval since BCG for most deaths, might obscure any benefits.

**Funding:**

British Leprosy Relief Association (LEPRA); Wellcome Trust.

## Introduction

There is considerable interest in the non-specific beneficial effects of the tuberculosis vaccine, BCG, on response to other infections. Ecological studies and BCG's immunological effects have suggested that BCG vaccination might be of use in the COVID-19 pandemic.^1^, ^2^, ^3^ A search of ClinicalTrials.gov shows that there are 17 randomised controlled trials comparing BCG versus placebo to reduce incidence or severity of COVID-19, aiming to recruit several thousand participants.

Most of the evidence of non-specific benefit of BCG vaccination comes from studies of all-cause mortality in neonates and young children receiving their first BCG vaccination, with very scarce data in adults.^1^, ^4^ Yet, for the COVID-19 trials, the participants are adults, who will often have received a BCG vaccination in the past.

In populations in which a high proportion of deaths are due to infections, any beneficial effect of BCG vaccination on all-cause mortality might be seen in older children and adults, as well as in young children. Because those who receive or do not receive a BCG vaccination in routine programmes are likely to have different socioeconomic backgrounds and health-seeking behaviour,^5^ and so different mortality risks, findings from observational data that might generate hypotheses on the non-specific effects of BCG vaccination require substantiation by randomised trials.^4^

**Figure 1:**
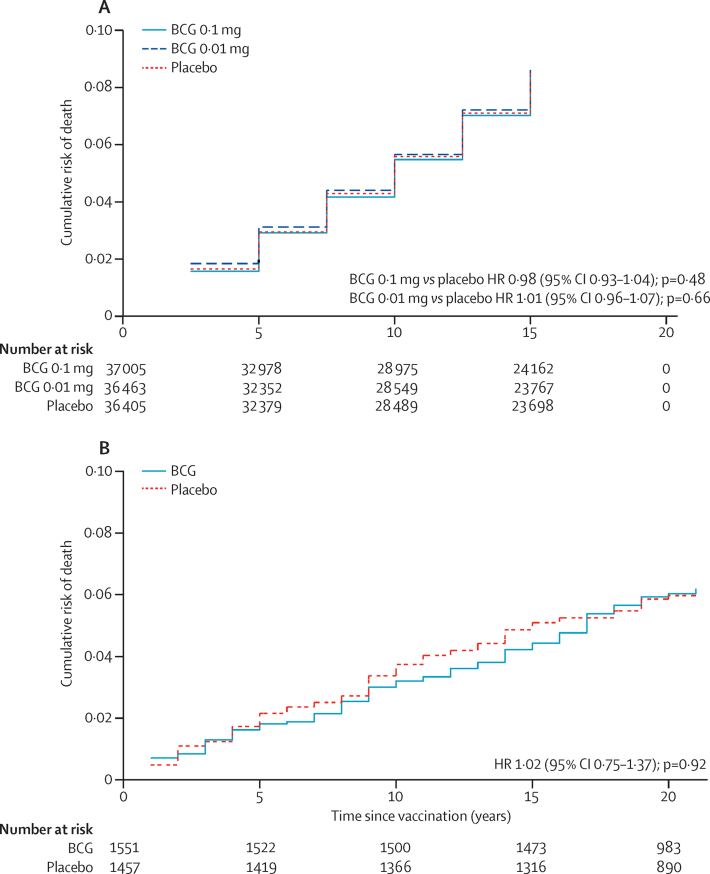
Mortality over time in published trials of BCG versus placebo (A) In Chingleput, south India (follow-up every 2·5 years). ^7^ (B) Among Native Americans, USA (annual follow-up).^9^ Nelson Aalen plots produced from summary data reported. HR=hazard ratio.

Of the trials included in a systematic review of the effect of BCG vaccination on tuberculosis,^6^ only four trials that included vaccination in individuals older than 5 years reported any data on mortality. None of the four trials show any effect of BCG vaccination on non-tuberculosis mortality (appendix pp 2–3).^7^, ^8^, ^9^, ^10^ In Chingleput, south India,^7^ and among Native Americans in the USA,^9^ data were shown (but not analysed), allowing survival curves to be constructed. There was no evidence in either trial of a difference in non-tuberculosis mortality between vaccinated and non-vaccinated groups at any time after vaccination (figure 1). Two further trials provide evidence on mortality. In a trial in Algiers in 1935, children were allocated by odd or even numbers at birth registration to receive oral BCG at birth, and at 1, 3, and 7 years of age, or no BCG. All-cause mortality was similar in the two groups during infancy, but lower in the multiply vaccinated group at older ages (appendix pp 2–3).^11^ Interim results from a recent small trial of BCG vaccination in older people were also consistent with protection but with wide CIs (appendix pp 2–3).^12^

**Research in context**

**Evidence before this study**

BCG vaccination is used to prevent tuberculosis, but there is increasing evidence that it can have non-specific beneficial effects. A systematic review has shown a reduction in all-cause mortality in very young children, and other reviews have shown wide-ranging effects on the immune system. To find BCG trials that reported on mortality in older age groups, we searched PubMed (on July 7, 2020) with no language restrictions to find the most recent systematic review of the effect of BCG on tuberculosis (searching on “BCG” and “trial”), and followed up further papers from the included trials for mortality outcomes. BCG has been suggested as a possible intervention for COVID-19, but there are very few data on the effects of BCG vaccination or revaccination on mortality in adults.

**Added value of this study**

In an extended follow-up of a large, double-blind, randomised trial in northern Malawi of BCG vaccination versus placebo in individuals of all ages who had a BCG scar we found no evidence of any effect of BCG revaccination on mortality. This is in line with data from other large trials including adults, which showed no effect of BCG vaccination on non-tuberculosis mortality.

**Implications of all the available evidence**

There is little evidence that a repeat BCG vaccination reduces non-tuberculosis mortality in adults. Any non-specific immune modulating effects of BCG do not seem to have large or long-term effects in these age groups.

In northern Malawi, a large trial of repeat BCG vaccination versus placebo in individuals of all ages with BCG scars recruited participants in 1986–89.^13^ This trial showed that BCG revaccination provided 49% protection against leprosy, but no protection against tuberculosis. Using information from two periods of active follow-up, we assessed all-cause mortality in individuals who received BCG revaccination or placebo.

## Methods

### Study design

The Karonga Prevention Trial, in Karonga District, northern Malawi, was a population-based, double-blind, randomised controlled trial, established to assess the effects of BCG vaccination, with or without killed *Mycobacterium leprae*, on the incidence of leprosy and tuberculosis. The methods are described in detail elsewhere.^14^, ^15^ The trial protocol was approved in 1985 by the Health Sciences Research Committee of the Malawi Ministry of Health, the Standing Committee on Research in Human Subjects of WHO, and the Ethics Committee of the London School of Hygiene & Tropical Medicine. Follow-up of the population has been approved in the context of other studies by the Health Sciences Research Committee of the Malawi Ministry of Health and the Ethics Committee of the London School of Hygiene & Tropical Medicine.

### Participants

Recruitment took place house-to-house from January, 1986, to November, 1989, throughout Karonga District, following meetings with traditional authorities and village headmen. The trial was explained to each household and informed, oral consent sought. All residents were eligible except individuals younger than 3 months or born before 1914, and individuals with evidence of past or current leprosy or tuberculosis, severe malnutrition, or other severe illness. Older individuals could request to be included.

### Randomisation and masking

All participants were examined for BCG scars as evidence of previous BCG vaccination. Individuals without BCG scars were randomly assigned to receive BCG vaccination alone or with killed *M leprae*. Participants who had BCG scars were randomly assigned to receive BCG vaccination alone, placebo, or BCG and killed *M leprae.* Because no participants without BCG scars received placebo, the data reported here are restricted to scar-positive individuals who received BCG vaccination alone or placebo.

The BCG and placebo (dextran matrix of the BCG vaccine) were provided by and manufactured by Glaxo, UK, in identical multidose vials. Random order codes for the vials were provided by WHO-appointed trial monitors. Randomisation was thus by small group rather than individual. Open vials were destroyed at the end of each day, and a mean of 6·9 doses (range 1–11) were used per vial. The main results were published in 1996, but the investigators remained masked to the vaccine codes until 2019.^13^

### Outcomes and follow-up

The trial was designed to assess the outcomes of leprosy and tuberculosis but for this analysis, the outcome was all-cause mortality among all participants who were included in active follow-up that occurred in two periods. Cause of death was not known for most of the participants.

For the follow-up study reported here, all individuals who had been living in four designated areas to the north of the district during the vaccine trial were sought in four different house-to-house surveys in 1991–94. These areas were selected because they had relatively high leprosy incidence. Participants who had left the areas were traced elsewhere in the district. If they had died, the year of death was recorded. If they were not found, the year they left the district was recorded.^13^, ^14^

A demographic surveillance area was established in the southern part of the district in 2002–04 and continues to date. This area was chosen because it had some data available on HIV prevalence in earlier surveys and was close to the project campus. Individuals who had been living in the area during the trial and were not known (from other studies in the district) to have died or moved, were actively sought as part of the baseline census. For those who were reported to have died or moved, the year of death or leaving was recorded. Those living in the demographic surveillance area are monitored through monthly reporting of deaths and annual censuses.^16^, ^17^ For the subgroup of participants from the southern area who were included in the demographic surveillance, cause of death is available from verbal autopsies done with household members.^18^ Informed consent was obtained for the follow-up studies.

### Statistical analysis

We used survival analysis to assess the effect of BCG revaccination on mortality, with p values calculated by the log-rank method, and hazard ratios (HRs) calculated using Cox regression. We estimated mortality from the date of vaccination, censoring individuals on the date when they were last known to be alive (or Dec 31, 2018, for those alive and under surveillance in the southern area). For individuals who had died or left, the date of death or departure was taken as the midpoint of the reported year, or halfway between the vaccination date and the end of the year for those who were reported to have died or left in the year in which they were vaccinated. For those in the demographic surveillance, exact dates were used. Analyses were done separately for the two different follow-up areas because of the different follow-up periods and procedures. Using an intention-to-treat approach, for each area we assessed the effect of BCG revaccination on mortality overall, in different age groups at vaccination (<5 years, 5–14 years, 15–24 years, and 25–75 years), and by sex.

For the subgroup included in the demographic surveillance, a further analysis considered cause-specific mortality, estimated from the time the participants were first seen in the demographic surveillance. All analyses used STATA/IC version 16.1.

### Role of the funding source

The funder of the study had no role in study design, data collection, data analysis, data interpretation, or writing of the report.

## Results

**Figure 2:**
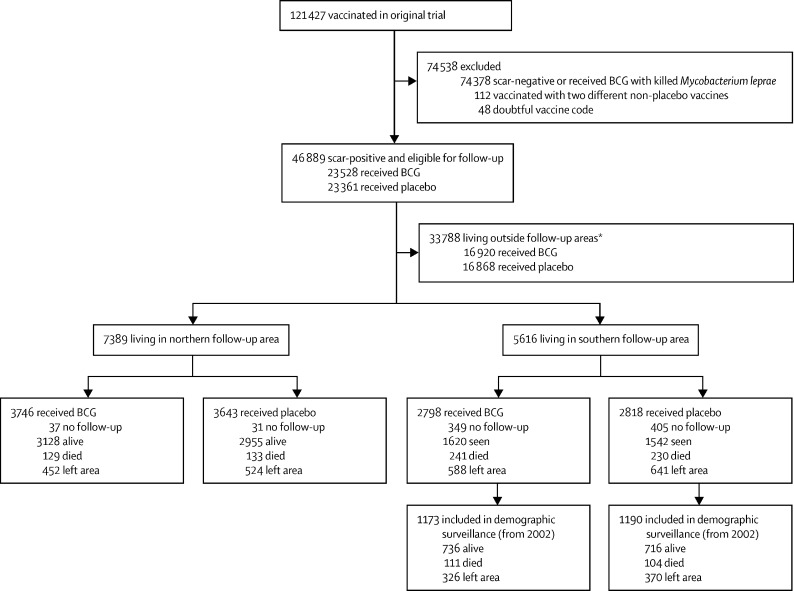
Flow diagram of follow-up of Karonga Prevention Trial, Malawi Alive=alive and seen when sought in survey. Died=reported dead when sought. Left area=reported to have left the district (in northern surveys) or the area (in southern survey). Seen=seen at least once after vaccination. *28 participants who received BCG and 32 who received placebo were included in both follow-ups because they had moved during the trial.

For the original study, 121 427 participants were recruited from January, 1986, to November, 1989. As previously reported, 5757 individuals were not eligible and 5835 refused to participate and were therefore not vaccinated as part of the trial.^13^ Individuals included in the follow-up analyses are summarised in figure 2. The characteristics of those included in the trial and in the two follow-up areas are shown in the table, and the characteristics of those who were not seen after vaccination or who were recorded as having left during the follow-up are summarised in the appendix (pp 4–5).

**Table:** Baseline characteristics of those in the Karonga Prevention Trial, overall and among those with follow-up information in the two areas

	**Total vaccinated**		**Northern follow-up population**		**Southern follow-up population**	
	Received BCG (n=23 528)	Received placebo (n=23 361)	Received BCG (n=3709)	Received placebo (n=3612)	Received BCG (n=2449)	Received placebo (n=2413)
**Age at vaccination, years**						
<5	3598 (15·3%)	3542 (15·2%)	610 (16·5%)	604 (16·7%)	269 (11·0%)	249 (10·3%)
5–14	9692 (41·2%)	9704 (41·5%)	1521 (41·0%)	1516 (42·0%)	1043 (42·6%)	1030 (42·7%)
15–24	5911 (25·1%)	5926 (25·4%)	830 (22·4%)	822 (22·8%)	705 (28·8%)	684 (28·4%)
25–75	4327 (18·4%)	4189 (17·9%)	748 (20·2%)	670 (18·6%)	432 (17·6%)	450 (18·7%)
**Sex**						
Male	11 670 (49·6%)	11 545 (49·4%)	1859 (50·1%)	1833 (50·8%)	1199 (49·0%)	1191 (49·4%)
Female	11 858 (50·4%)	11 816 (50·6%)	1850 (49·9%)	1779 (49·3%)	1250 (51·0%)	1222 (50·6%)

**Figure 3:**
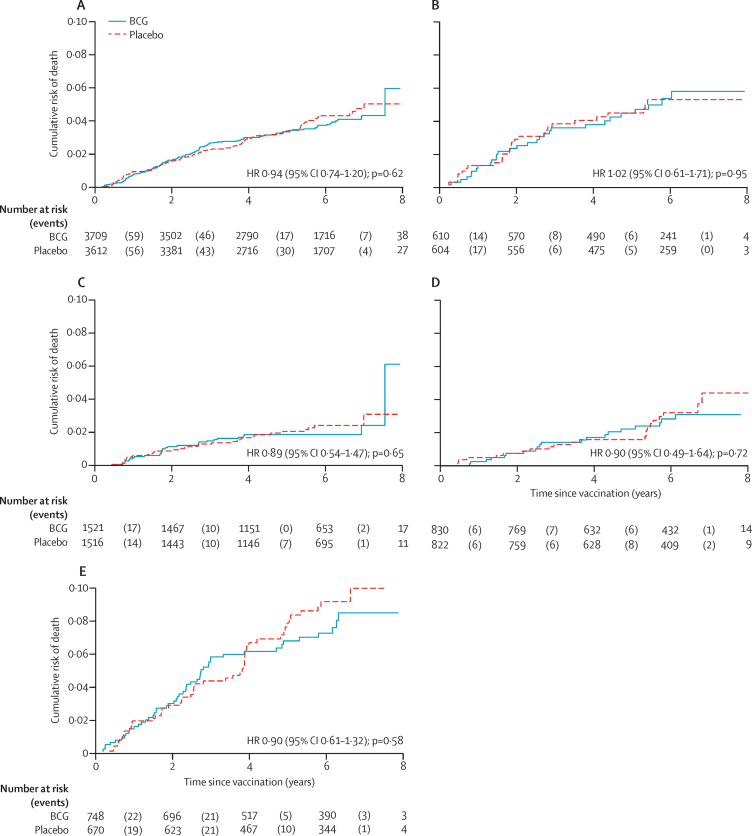
Mortality by BCG revaccination status in northern area of Karonga District, to 1994 (A) All age groups. (B) Population younger than 5 years at vaccination. (C) Population aged 5–14 years at vaccination. (D) Population aged 15–24 years at vaccination. (E) Population aged 25–75 years at vaccination. HR=hazard ratio.

The areas covered in the northern surveys in 1991–94 included 3746 (15·9%) of the 23 528 individuals who had received BCG and were eligible for follow-up and 3643 (15·6%) of the 23 361 who had received placebo and were eligible for follow-up. Follow-up information was available at some point after vaccination for 7321 (99·1%) of the 7389 participants living in the northern area during the follow-up period (figure 2). In the northern area, there was no difference in mortality between those who received BCG and those who received placebo: there were 129 deaths per 19 694 person-years at risk in those who had received BCG (6·6 deaths per 1000 person-years at risk [95% CI 5·5–7·8]) and 133 deaths per 19 111 person-years at risk in those who had received placebo (7·0 deaths per 1000 person-years at risk [95% CI 5·9–8·2]; HR 0·94 [95% CI 0·74–1·20]; p=0·62). There was no difference between the BCG revaccination and placebo groups in any age group (likelihood test for interaction by age group, p=0·98; figure 3). There was also no difference by sex (likelihood test for interaction p=0·74; appendix p 6).

**Figure 4:**
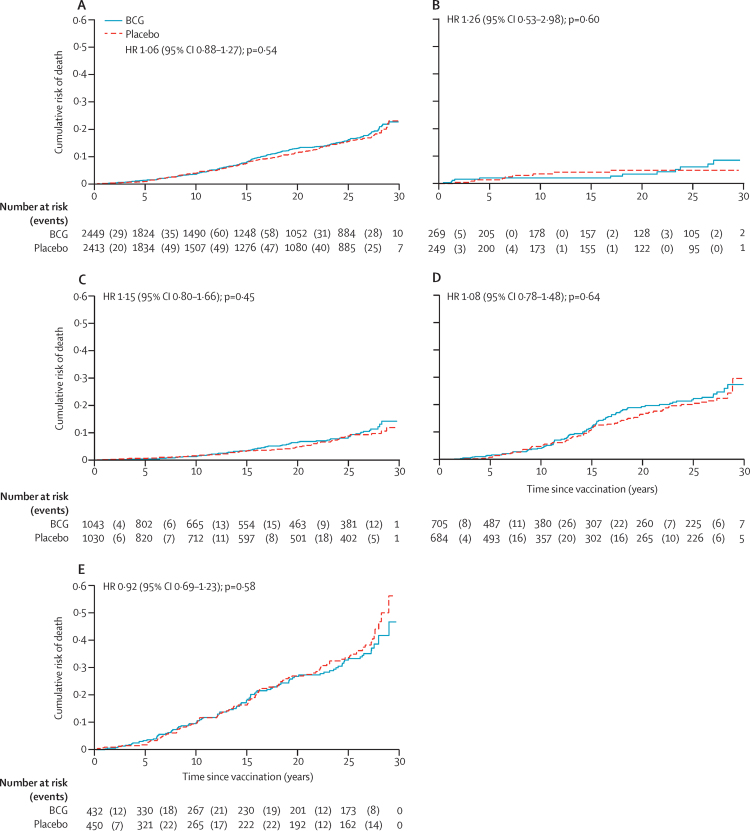
Mortality by BCG revaccination status in southern area of Karonga District, to 2018 (A) All age groups. (B) Population younger than 5 years at vaccination. (C) Population aged 5–14 years at vaccination. (D) Population aged 15–24 years at vaccination. (E) Population aged 25–75 years at vaccination. HR=hazard ratio.

The demographic surveillance site covered an area in the southern part of the district. Of the 46 889 BCG scar-positive individuals who received BCG or placebo, 5616 (12·0%) were living in this area during the trial recruitment. We had follow-up information at some point after vaccination for 4862 (86·6%), nearly half of whom were seen in the demographic surveillance (figure 2). There was no difference in mortality by vaccine status in the southern area: there were 241 deaths per 38 399 person-years at risk in those who received BCG (6·3 deaths per 1000 person-years at risk [95% CI 5·5–7·1]) compared with 230 deaths per 38 676 person-years at risk in those who received placebo (5·9 deaths per 1000 person-years at risk [95% CI 5·2–6·8]; HR 1·06 [95% CI 0·88–1·27]; p=0·54). There was no difference in survival between BCG and placebo groups in any age group (likelihood test for interaction by age group p=0·75; figure 4) or by sex (likelihood test for interaction p=0·24; appendix p 4).

In the subgroup of the southern population who were still alive and living in the area during the period of demographic surveillance (2002–18), there were no differences between the BCG and placebo groups in all-cause mortality, mortality excluding external causes, mortality due to communicable disease, or mortality due to communicable diseases other than HIV/AIDS and tuberculosis (appendix p 7).

## Discussion

We found no evidence of an effect of revaccination with BCG on all-cause mortality, either overall or in any age group. Importantly, we also found no evidence of any detrimental effect. A high proportion of individuals were followed up at least once after vaccination, and as the data come from a double-blind, randomised controlled trial, confounding and bias (eg, by socioeconomic status or health seeking behaviour) should be minimal.

We relied on reported years of death and of departure from the area, so there might have been some inaccuracy in dates of events (for both BCG and placebo groups). It is possible that some deaths were missed among those with incomplete follow-up information. In the northern area, almost everyone had some follow-up information, but the proportion who were reported to have left the district during follow-up was higher in the placebo group than the BCG group (figure 2; appendix p 4). In the southern area, the proportion with no follow-up and the proportion who were reported to have left the area during follow-up were both slightly higher in the placebo group (figure 2; appendix p 4–5). In both areas, the imbalance in those who had left between the placebo and BCG groups was only seen in those vaccinated as children (younger than 5 years in both areas, and also aged 5–14 years in the northern area; appendix pp 4–5).

If those who were not seen for follow-up after revaccination or were reported to have left the area were more likely to have died, then it is possible that this could have masked some non-specific beneficial effect of repeat BCG vaccination on mortality in those revaccinated as children. We analysed data from the northern and southern areas separately because of the different follow-up periods and methods. The data from the northern area are likely to be more accurate than those from the southern area because of the shorter period of recall for dates, and a more exhaustive search for those in the trial. In the southern area, deaths could have been missed among those with no records following vaccination, but the proportion with no follow-up was only slightly higher in the placebo group than in the vaccination group (appendix p 5). The most intensive follow-up was from 2002 among those in the demographic surveillance area in the south. Migration and death should have been reported accurately in this group so the slightly higher proportion of placebo recipients than BCG recipients who were reported to have left is likely to be due to chance, not misclassification. Differences in follow-up are very unlikely to explain the absence of an association between repeat BCG vaccination and mortality in adults.

Neonates were not included in this study and there is little evidence in other studies of benefit from BCG on all-cause mortality in children vaccinated at older ages,^4^ or in adults (appendix pp 2–3).^7^, ^8^, ^9^, ^10^ In some studies in children, the order of vaccines has been found to influence the effect on mortality, and subsequent vaccines could theoretically influence the results.^19^ However, in our trial, very few individuals would have received subsequent vaccines. The first polio national immunisation day was in 1996^20^ and, as these target children up to 5 years old, they would not have included the BCG vaccination trial population. The first measles vaccination campaign was in 1998 and targeted individuals up to 15 years old,^21^ so would only have included the very youngest age group in the southern analysis.

It has been suggested that the non-specific effects of BCG vaccination might vary by strain, and that this could explain the reduction in infant all-cause mortality seen in trials in Guinea-Bissau using BCG-Denmark, and not in India using BCG-Russia.^22^ The strain used in this study, BCG-Glaxo, is genetically close to the BCG-Denmark strain. In a comparison between Danish and Glaxo BCG strains in UK schoolchildren the immunogenicity was similar, and BCG-Glaxo produced larger scars, which might correlate with non-specific effects.^22^, ^23^

The duration of any beneficial non-specific effects of BCG vaccination is unknown. The data from Chingleput, south India, and from Native Americans in the USA both show no difference in non-tuberculosis mortality between the vaccine groups at any time since vaccination (figure 1).^7^, ^9^ Our trial found similar null results, throughout follow-up and in all age groups (Figure 3, Figure 4).

The study size was limited by the data available. Based on the rates observed in the placebo group and the median follow-up times, we had 80% power to detect HRs of less than 0·68 in the northern area and less than 0·74 in the southern area, at the 5% level of significance.

All-cause mortality is likely to be harder to influence by immune modulation in adults than in young children because a smaller proportion of deaths will be due to acute infections in the adult population than among children. Cause of death was not known for most of the population in this study. In the subgroup for whom we had this information, there was no evidence of protection against communicable disease deaths, but numbers were small, and this was a select group who had already survived at least 13 years since vaccination. In the wider demographic surveillance population from 2004–09, we have previously shown that the number of deaths in adults due to communicable disease was 419 (46·3%) of 905 deaths, with 364 (40·2%) due to non-communicable disease and only 47 (5·2%) attributable to external causes (75 [8·3%] unknown).^18^ Over that period, the proportion of deaths due to HIV/AIDS decreased sharply (from 42% to 17% of deaths) following the roll-out of antiretroviral therapy from 2005, but the rates of death due to non-communicable and communicable disease other than HIV/AIDS were approximately constant.^18^ In the northern area, the proportion of deaths due to HIV/AIDS is likely to have been smaller than in the southern area because the follow-up period was before the peak of the HIV epidemic.

There is little direct evidence of a non-specific effect of BCG vaccination on morbidity in adults. In a trial in South African adolescents, it was noted that BCG revaccination was associated with a lower incidence of upper respiratory tract infections, but this was one of a long list of possible adverse events recorded, not a prespecified outcome of the trial.^24^ Two small trials, which lack detailed reporting, suggest that BCG vaccination confers protection against pneumonia and upper respiratory tract infection in older people.^25^, ^26^ In 2020, an interim analysis of a randomised controlled trial in older people found reduced infections (HR 0·55 [95% CI 0·31–0·97]) over 12 months.^12^

Several reviews have catalogued the effects of BCG vaccination on different aspects of the immune system, including in in-vitro and mouse studies.^27^, ^28^, ^29^ Many results are not consistent between studies, but BCG vaccination seems to increase interferon-γ, especially after challenge with other antigens.^27^ More recent studies suggest that BCG might act primarily through trained innate immunity.^28^, ^29^, ^30^ Challenge studies with other infections have had mixed results—eg, lower viraemia and no difference in antibody concentrations following yellow fever vaccination^30^ but more severe symptoms following *Plasmodium falciparum* infection^31^—and studies show considerable interindividual variability in responses.

Although there is little evidence of an effect of BCG vaccination on mortality in adults in our results or elsewhere, it is well known that the protective effects of BCG against tuberculosis vary by setting,^5^ and there is strong evidence from other studies that it does have immunomodulatory effects. We await the results of the COVID-19 trials with interest.

## Data sharing

On publication, de-identified individual participant data that underlie the results reported in the Article will be made available via Datacompass (https://datacompass.lshtm.ac.uk/1872/). Proposals should be directed to chifundo.kanjala@lshtm.ac.uk; to gain access, data requestors will need to sign a data access agreement.

## Declaration of interests

All authors declare no competing interests.
