# Supplementary material for: The effect of BCG revaccination on all-cause mortality beyond infancy: 30-year follow-up of a population-based, double-blind, randomised placebo-controlled trial in Malawi
Source: Lancet Infect Dis. 2021 Nov;21(11):1590–7. doi: 10.1016/S1473-3099(20)30994-4 (PMC8550897; doi:10.1016/S1473-3099(20)30994-4)
Supplement: Supplementary appendix [file mmc1.pdf]

THE LANCET
Infectious Diseases

Supplementary appendix

This appendix formed part of the original submission and has been peer reviewed. We post it as supplied by the authors.

Supplement to: Glynn JR, Dube A, Fielding K, et al. The effect of BCG revaccination on all-cause mortality beyond infancy: 30-year follow-up of a population-based, double-blind, randomised placebo-controlled trial in Malawi. *Lancet Infect Dis* 2021; published July 5. https://doi.org/10.1016/S1473-3099(20)30994-4.

The effect of BCG revaccination on all-cause mortality beyond infancy: 30-year follow-up of a population-based, double-blind, randomised placebo-controlled trial in Malawi

Appendix

Judith R Glynn,[1] Albert Dube,[2] Katherine Fielding,[1] Amelia C Crampin,[1,2] Karonga Prevention Trial Group,[3] Chifundo Kanjala, [1,2] Paul EM Fine[1]

1. Faculty of Epidemiology and Population Health, London School of Hygiene & Tropical Medicine, UK
2. Malawi Epidemiology and Intervention Research Unit, Malawi
3. Members:

Original Karonga Prevention Trial Group:
Coordinator—P E M Fine (London School of Hygiene and Tropical Medicine, London, UK).
Fieldwork directors—J M Pönnighaus (LEPRA, Chilumba, Malawi), D K Warndorff (LEPRA).
Group participants—Field medical officers: P J K Gruer, S Oxborrow,P D P Pharoah (LEPRA); histopathologists: S B Lucas (University College Hospital Medical School, London, UK), A C McDougall (Slade Hospital, Oxford, UK); mycobacteriologist: P A Jenkins (UKPHLS Mycobacterium Reference Laboratory, Cardiff, UK); senior paramedical staff: D Chavula, G Msiska, E Msosa, M Munthali, B Mwamondwe, D Ng'oma, P Nkhosa, H Phiri, M Simfukwa (LEPRA); senior interviewers: A Chihana, D Kawaonja, S Malema, V Mwinuka (LEPRA); senior laboratory staff: P Mkandawire, S Nyasulu, H Tegha (LEPRA); senior data managers: M Kalambo, S Kileta, M Simwaka (LEPRA); computing staff: L Bliss, J Saul (London School of Hygiene and Tropical Medicine); statisticians: N Maine, J A C Sterne (London School of Hygiene and Tropical Medicine); epidemiologist: J R Glynn (London School of Hygiene and Tropical Medicine); Clinical monitor: D Bell (Liverpool School of Tropical Medicine, Liverpool, UK); WHO monitors: D Clayton (MRC Biostatistics Unit, Cambridge), UK, M C Pike (ICRF Cancer Epidemiology Unit, Oxford, UK).
Diagnostic review team—I Cree (Institute of Ophthalmology, London, UK), K Desikan (MG Institute of Medical Sciences, India), M D Gupte (Indian Council of Medical Research, Avadi, India), R R Jacobson (USPHS Hansen's Disease Centre, Carville, Louisiana, USA), D S Nyangulu (Ministry of Health, Lilongwe, Malawi).
Data monitoring committee—D Clayton, J Darbyshire (MRC HIV Clinical Trials Unit, London, UK), M D Gupte, R Peto (ICRF/MRC/BHF Clinical Trials Service Unit, Oxford, UK).

Contributors to demographic surveillance:
(excluding those who were part of the original trial group)
E Banda, K Branson, FM Chimbwandira, M Chihana, Z Chirwa, AC Crampin, A Dube, S Floyd, N French, S Geis, H Jabu, S Jaffar, A Jahn, L Kachiwanda, F Kalobekamo, AM Kaonga, A Katsulukuta, C Katundu, M Kayange, N Kayuni, A Khunga, M Kondowe, O Koole, K Kranzer, M Luhanga, S Mboma, N McGrath, E McLean, RE Mkisi RE, A Molesworth, A Msona, F Munthali, H Mvula, J Mwafilaso, C Mwafulirwa, D Mwagomba, E Mwaiyeghele, O Mwanyongo, F Mwaungulu, L Mwaungulu, O Mwiba, P Mzumara, BM Ngwira, MH Ngwira, MJ Nyirenda, A Price, T Tafatatha, B Zaba

Table S1. Previous trials of BCG vs placebo that have included individuals aged more than 5 years and have reported mortality

Study	Vaccination		Follow-up	Tuberculin response at recruit-ment	Allocation	Outcome	Vaccine group	No. of deaths	Rate/1000 pyar (or risk%), with 95% CI	Relative risk (95% CI)
	Age	Year								
[a] Chingleput, South India[1]	All ages > 1 month	1968-71	15 years	Negative (0-7mm)	Individual random-isation	Death exclud-ing culture positive TB	BCG 0.1mg<br>BCG 0.01mg<br>Placebo	2531<br>2572<br>2537	5.61 (5.39-5.83)<br>5.79 (5.57-6.02)<br>5.72 (5.50-5.95)	0.98 (0.93-1.04)<br>1.01 (0.96-1.07)<br>1
[b] Native Americans, USA[2]	0-19 years	1936-8	20 years	Negative	Alternate	Death not due to TB	BCG<br>Placebo	91<br>82	3.02 (2.46-3.71)<br>2.99 (2.40-3.71)	1.02 (0.75-1.37)<br>1
						Death not TB or violence	BCG<br>Placebo	46<br>42	1.53 (1.14-2.04)<br>1.53 (1.13-2.07)	1.0 (0.66-1.51)<br>1
[c] MRC trial, UK[3]	14-15 years	1950-52	15 years	Negative	Individual random-isation	Death not due to TB	BCG<br>Placebo	93<br>92	0.477 (0.389-0.585)<br>0.456 (0.372-0.559)	1.05 (0.78-1.40)<br>1
						Death not TB or violence	BCG<br>Placebo	40<br>48	0.196 (0.144-0.267)<br>0.249 (0.188-0.330)	0.79 (0.52-1.20)<br>1
[d] Institution USA[4]	All ages	1948	12 years	Negative	Not stated	Death not due to TB	BCG<br>"controls"	65<br>70	12.2% (9.58-15.3)<br>14.2% (11.2-17.6)	0.86 (0.63-1.18)<br>1
[e] Algiers, Algeria[5]	0,1,3,7 years	1935-54	Age 7 -11 years	Not stated	Odd or even no.	Death all causes	Oral BCG x4<br>Placebo	87<br>114	0.72 (0.58-0.89)<br>1.08 (0.89-1.30)	0.66 (0.50-0.88)<br>1
[f] Post-hospital, Greece[6]	≥ 65 years	2019-20	1 year	Negative IGRA	Individual random-isation	Death all causes (no TB diagnosed)	BCG<br>Placebo	10<br>14	13.9% (6.9-24.1)<br>17.9% (10.2-28.3)	0.68 (0.30-1.54)<br>1

Footnotes to table S1

TB=tuberculosis; CI= confidence interval; pyar=person years at risk; IGRA=interferon gamma release assay

BCG strains used: Chingleput - Danish/Copenhagen/1331, Paris/Pasteur-1173 P2; Native Americans - Phipps/Pasteur 317, Pasteur 575; MRC- Danish/Copenhagen,; Institution – not specified; Algiers – not specified; Greece - Intervax Bulgaria strain.

[a] Active follow-up every 2.5 years. Rate and rate ratio calculated from published data
[b] Active follow-up annually. Rate and rate ratio calculated from published data. Note: mortality due to tuberculosis was much higher in the placebo group than in the BCG group
[c] Confidence intervals and rate ratio estimated from published data
[d] Institution for people with learning difficulties. Randomisation method not stated and not clear if groups were comparable. 522/531 BCG vaccinated were tuberculin tested. 258/262 who remained negative were revaccinated. Mortality risk and risk ratio estimated assuming those discharged from institution during follow-up (117 BCG recipients and 88 controls) were alive. Excluding those who were discharged gives a risk ratio of 0.91 (95% CI 0.67-1.24).
[e] Rate and rate ratio calculated from published data. Age group selected to estimate the effect of revaccination at age 7 years
[f] Confidence intervals for risks calculated from published data. Hazard ratio from the paper. It is likely this population had had previous BCG vaccination.

References for table S1

1. Tuberculosis Research Centre (ICMR) Chennai. Fifteen year follow up of a trial of BCG vaccines in south India for tuberculosis prevention. *Indian J Med Res* 1999; **110**: 56-69.
2. Aronson JD, Aronson CF, Taylor HC. A twenty-year appraisal of BCG vaccination in the control of tuberculosis. *Archives of Internal Medicine* 1958; **101**: 881-93.
3. Medical Research Council Tuberculosis Vaccines Clinical Trials Committee. BCG and vole bacillus vaccines in the prevention of tuberculosis in adolescence and early adult life. *Bull World Health Organ* 1972; **46**(3): 371-85.
4. Bettag OL, Kaluzny AA, Morse D, Radner DB. Bcg Study at a State School for Mentally Retarded. *Dis Chest* 1964; **45**: 503-7.
5. Sergent E, Catanei A, Ducros-Rougebief H. Prémunition antituberculeuse par le BCG. Campagne contrôlée poursuivie à Alger depuis 1935. Troisième note. *Arch Inst Pasteur Alger* 1960; **38**: 131-7.
6. Giamarellos-Bourboulis EJ, Tsilika M, Moorlag S, et al. Activate: Randomized Clinical Trial of BCG Vaccination against Infection in the Elderly. *Cell* 2020; **183**: 1-9.

Exploration of incomplete follow-up by vaccine group, age at vaccination and area.

Those with incomplete follow-up are a combination of those with no follow-up and those who were recorded as "left" during the follow-up. Note that those who "left" had new areas of residence recorded (after the first northern survey). They are assumed to have been alive at the date that they were reported to have "left".

As described in the main paper, because multi-dose vials were used, the randomisation was by small group rather than by individual, with an average of 7 individuals per vial. Household members and close neighbours were therefore likely to share a vial, leading to clustering of who got placebo and who got BCG. This is not an issue for the main analysis, because there is little clustering of deaths, but since whole households are likely to migrate together, clustering becomes important in assessing any differences between BCG and placebo groups. The analyses below therefore take account of clustering by vaccine vial, using robust standard errors.

Northern areas

In the northern areas everyone was sought during the follow-up so the last information was obtained during the follow-up survey. However 68 participants (37 BCG, 31 placebo) were recorded as having "left" the same year as the vaccination, so they are not included in the mortality analysis (Figure 2), or in the hazard ratios shown below.

Table S2: Proportion of participants in northern areas who were reported to have left, by vaccine group and age at vaccination, with hazard ratios for leaving.

	BCG	%	Placebo	%	HR placebo vs BCG	*p*
All	489/3746	13.1	555/3643	15.2	1.19 (1.02-1.39)	0.024
Age (years)						
< 5	64/614	10.4	92/610	15.1	1.46 (1.02-2.08)	0.038
5-14	169/1533	11.0	207/1526	13.6	1.26 (1.01-1.59)	0.045
15-24	172/843	20.4	178/831	21.4	1.08 (0.86-1.36)	0.51
25+	84/756	11.1	78/676	11.5	1.06 (0.75-1.48)	0.75

(Wald test for interaction by age: p=0.39)

Southern area

In the southern area most (97.1%, 732/754) of the individuals who had no follow-up had no record after the vaccination record. The remaining 22 have a later record but were recorded as having "left" around the time of vaccination. A further 1229 individuals were reported to have "left" during the subsequent 30 years.

We have looked separately at the risk for no follow-up (using risk ratios) and for those who were reported to have left later (using hazard ratios to take duration of follow-up into account). Both analyses use robust standard errors, to take account of clustering by vaccine vial, as above. In the under 5s, the increase in risk of leaving is only seen after about 15 years of follow-up, during the demographic surveillance.

Table S3. Proportion of participants in southern area who had no follow-up or were reported to have left, by vaccine group and age at vaccination, with risk ratios (RR) for no follow-up, and hazard ratios (HR) for leaving.

	BCG	%	Placebo	%	Placebo vs BCG	*p*
**Risk of no follow-up**					**RR (95% CI)**	
All	349/2798	12.5	405/2818	14.4	1.15 (0.97-1.37)	0.11
Age (years)						
< 5	63/332	18.9	72/321	22.4	1.18 (0.85-1.65)	0.33
5-14	158/1201	13.2	191/1221	15.6	1.19 (0.94-1.50)	0.15
15-24	69/774	8.91	76/760	10.0	1.12 (0.81-1.55)	0.48
25+	59/491	12.0	66/516	12.8	1.06 (0.75-1.51)	0.73
**Risk of leaving**					**HR (95% CI)**	
All	588/2449	24.0	641/2413	26.6	1.09 (0.95-1.24)	0.21
Age (years)						
< 5	89/269	33.1	110/249	44.2	1.34 (0.99-1.80)	0.056
5-14	330/1043	31.6	343/1030	33.3	0.97 (0.82-1.14)	0.73
15-24	127/705	18.0	148/684	21.6	1.21 (0.95-1.54)	0.12
25+	42/432	9.72	40/450	8.89	0.97 (0.62-1.54)	0.91

(Wald test for interaction by age: *p*=0.95 for no follow-up, and p=0.18 for leaving)

Figure S1: Mortality by BCG re-vaccination status by area and sex, Karonga District, Malawi: (a) northern areas, female;(b) northern areas, male; (c) southern area, female; (d) southern area, male. Note different scales in northern and southern areas due to the different follow-up times

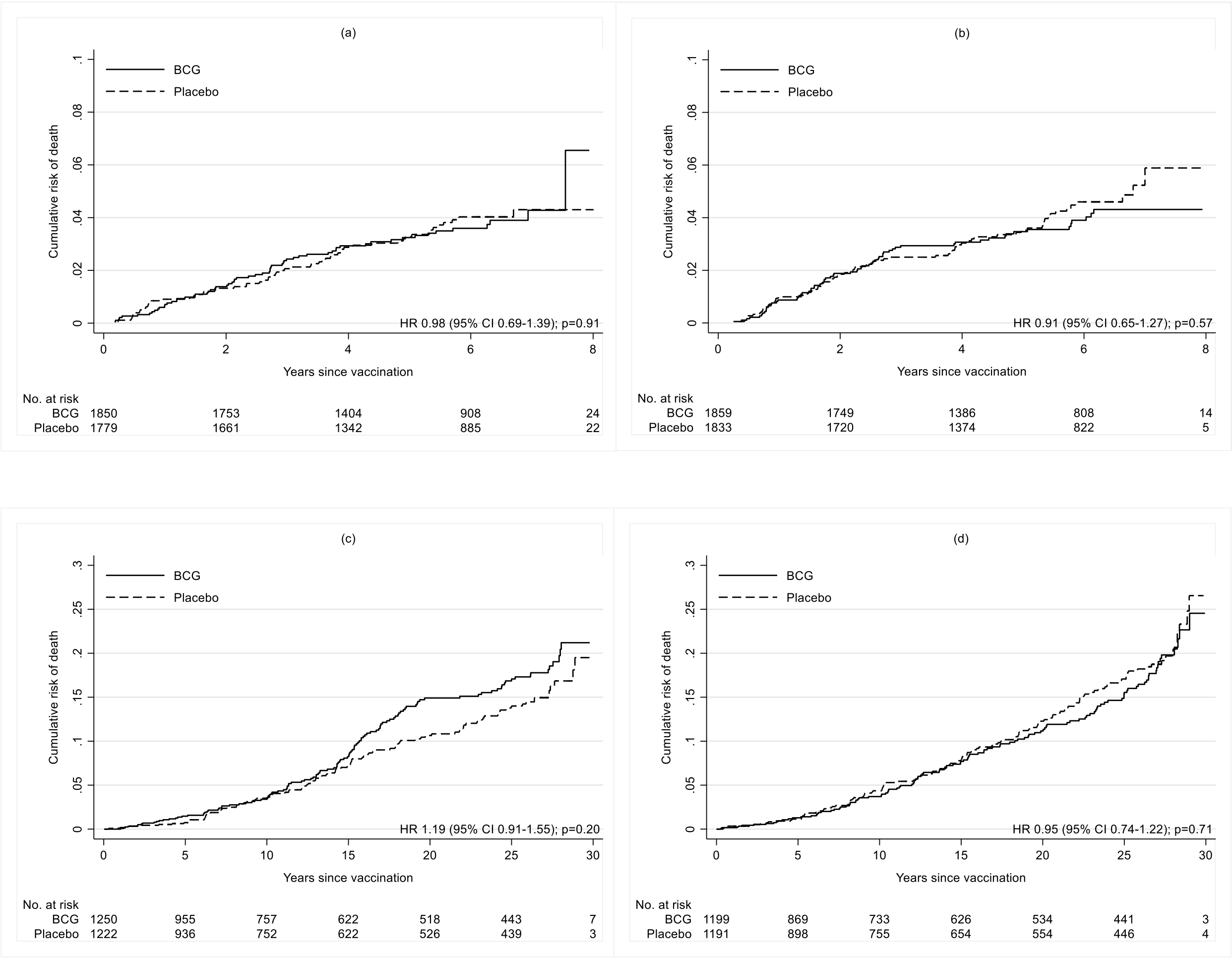

Table S4: Mortality from 2002 by BCG re-vaccination status in the population under demographic surveillance, by cause of death.

Vaccine trial participants were included in this analysis if they were living in the southern area during the vaccine trial, and were recorded as alive and living in this area at at least one point after the beginning of the demographic surveillance in 2002.

For this analysis participants were considered "at risk" from when they were first seen in the demographic surveillance. Cause of death was unknown or unclear for 11.6% (25/215) deaths.

		Deaths	Mortality/1000 pyar (95%CI)	HR (95%CI)	p
All cause mortality	BCG	109	8.95 (7.42-10.80)	1.04 (0.80-1.36)	0.75
	Placebo	104	8.58 (7.08-10.38)		
Excluding external and direct maternal	BCG	97	7.97 (6.53-9.72)	1.05 (0.79-1.40)	0.74
	Placebo	92	7.59 (6.18-9.31)		
Communicable disease only	BCG	60	4.93 (3.83-6.35)	1.34 (0.91-1.97)	0.15
	Placebo	45	3.71 (2.77-4.97)		
Communicable disease excluding HIV/AIDS/TB	BCG	9	0.74 (0.38-1.42)	0.82 (0.34-1.98)	0.65
	Placebo	11	0.91 (0.50-1.64)		

pyar = person years at risk (12,173 in the BCG group and 12,126 in the placebo group)

HR=hazard ratio, CI=confidence interval
